# Vanadium stimulates pepper plant growth and flowering, increases concentrations of amino acids, sugars and chlorophylls, and modifies nutrient concentrations

**DOI:** 10.1371/journal.pone.0201908

**Published:** 2018-08-09

**Authors:** Atonaltzin García-Jiménez, Libia Iris Trejo-Téllez, Dagoberto Guillén-Sánchez, Fernando Carlos Gómez-Merino

**Affiliations:** 1 Department of Plant Physiology, Colegio de Postgraduados Campus Montecillo, Texcoco, State of Mexico, Mexico; 2 Department of Soil Science, Laboratory of Plant Nutrition, Colegio de Postgraduados Campus Montecillo, Texcoco, State of Mexico, Mexico; 3 Xalostoc School of Higher Studies, Autonomous University of the State of Morelos, Xalostoc, Ayala, Morelos, Mexico; United States Department of Agriculture, UNITED STATES

## Abstract

Vanadium (V) can be absorbed by plants and regulate their growth and development, although contrasting effects have been reported among species and handling conditions. The objective of this work was to evaluate the beneficial effect of V on pepper plants (*Capsicum annuum* L.). The plants were grown in a hydroponic system with the application of four V concentrations (0, 5, 10, and 15 μM NH₄VO₃). Four weeks after the beginning of the treatments, growth, flowering, biomass, chlorophyll concentration, total amino acids, total soluble sugars, and nutrients were determined in leaves, stems, and roots. The application of 5 μM V increased plant growth, induced floral bud development, and accelerated flowering. The chlorophyll concentration varied according to the type of plant part analyzed. The concentrations of amino acids and sugars in leaves and roots were higher with 5 μM. With 10 and 15 μM V, the plants were smaller and showed toxicity symptoms. The K concentration in leaves decreased as the V dose increased (0 to 15 μM). However, 5 μM V increased the concentrations of N, P, K, Ca, Mg, Cu, Mn, and B, exclusively in stems. The application of 15 μM V decreased the concentrations of Mg and Mn in leaves, but increased those of P, Ca, Mg, Cu, and B in roots. We conclude that V has positive effects on pepper growth and development, as well as on the concentrations of amino acids and total sugars. V was antagonistic with K, Mg, and Mn in leaves, while in stems and roots, there was synergism with macro and micronutrients. Vanadium is a beneficial element with the potential to be used in biostimulation approaches of crops like pepper.

## Introduction

Vanadium (V) is a transition metal widely distributed in the Earth’s crust, where it may be found at a mean concentration of 20 to 120 mg kg^-1^ [1]; however, it has little mobility in soil, since less than 1% of the total V is extractable and leachable with water [2, 3]. The first studies identified V as an element highly toxic to plants [4], which decreased interest in evaluating its effect on cultivated species. It was not until the 1950s that Bertrand [5] observed that low concentrations of this element (10 ng V g^-1^ soil) could positively influence plant growth. Later studies indicated that due to the different states of oxidation of this element (which oscillate from -1 to +5), its toxicity to plants is associated with its pentavalent oxidation form (V^+5^), while the tetravalent form (V^+4^) can contribute to their development [6]. Although V^+4^ is the least toxic form of V, it is also considered the least mobile and the most predominant in soil [3].

V is a metal found in low concentrations in all plants, and its absorption is carried out through passive processes [7]. This element can act as a redox catalyzer in electron transportation in photosystems I and II, depending on the environmental conditions [8, 9]. With regard to its positive effects on plants, the application of doses under 0.05 mg L^-1^ V increased maize (*Zea mays*) production and kernel quality [10]. Moreover, the application of 250 ng mL^-1^ V increased height, number of leaves and flowers, and chlorophyll concentration in tomato plants (*Solanum lycopersicum*) [11]. In basil (*Ocimum basilicum*), the dry biomass of roots increased linearly with increasing V concentrations from 0 to 40 mg L^-1^ [12]. It has also been proven that V can counteract the negative effects of certain metals like Cu [13].

Another well-known function of V is its participation as a cofactor of the enzyme nitrogenase. V-dependent nitrogenase, discovered in *Azobacter vinelandii* [14], is found in a wide gamut of diazotrophic microorganisms, catalyzing the conversion of atmospheric dinitrogen into ammonia; and in contrast with molybdenum (Mo)-dependent nitrogenase, it also reduces carbon monoxide (CO) [15]. V interacts with other elements like P and Mo [16], and in the form of monomeric vanadate, it is structurally and electronically similar to phosphate (Pi), which enables it to participate in the inhibition and activation of enzymes that interact with phosphorylated substrates like phosphatases, ATPases, and phosphotransferases [15].

Besides the relevance of V in agriculture, in medicine V has been experimentally used as a treatment against diabetes mellitus [17]. In rats, the application of V decreases the glucose levels in blood [18], while in humans, the intake of V decreases insulin with no significant secondary clinical effects [19]. Apart from its anti-diabetic effect, it has been suggested that V can have pharmacological activity in the treatment of parasitic diseases, malign tumors, as well as bacterial and viral infections [20]. While an average daily diet may provide 5–20 μg V, in therapies the safe upper limit is 1.8 mg a day [21,22]. Higher doses could be toxic.

Therefore, the use of V in crop production can favor development as well as contribute to human health. In rice (*Oryza sativa*) treated with 20 mg L^-1^ V, 7.8 mg kg^-1^ V was found in the grains and 338.7mg kg^-1^ in leaves and stems. This indicates that V tends to accumulate in plant tissue [21]. In tomato, the application of 40 mg L^-1^ V produced fruits with 4.0 mg kg^-1^ V [23]. Given the different responses to different V concentrations applied, the need for further studies is evident in order to ensure optimum doses of this element as a biostimulant in different genotypes of cultivated plants.

Pepper (*Capsicum annuum*) is one of the most important crops in economic terms worldwide. Mexico is the second main world producer of this crop, besides being one of the centers of origin and diversification of this species. Despite pepper being grown all over the world and its uses as a vegetable, condiment, medicine, coloring, and source of essential vitamins, there are few studies on the use of beneficial elements to increase its productivity. Specifically, the effect of V in pepper production has not been studied to date, and its assessment is crucial in order to test if its application can stimulate some mechanisms that will improve plant performance [24]. The objective of the present work was to evaluate the effect of different V concentrations on growth, flowering, chlorophyll concentration, amino acids, sugars, and nutrients in pepper plants grown in a hydroponic system, in order to identify possible beneficial effects.

## Materials and methods

### Plant material and growth conditions

Pepper (*Capsicum annuum*) cv. Mysterio F1 seeds were germinated in polypropylene trays with 200 cavities using peat moss substrate (Theriault & Hachey Peat Moss Ltd., Growing Mix; Baie Sainte-Anne, New Brunswick, Canada). At 30 days after sowing, the seedlings were transplanted to 35 L plastic containers with Steiner nutrient solution at 20%, containing 1.8 mM Ca(NO_3_)_2_ 4H_2_O, 0.8 mM MgSO_4_ 7H_2_O, 0.2 mM KH_2_PO_4_, 0.6 mM KNO_3_, 0.6 mM K_2_SO_4_, 89.31 μM Fe, 42.37 μM Mn, 7.12 μM Zn, 39.98 μM B, 2.93 μM Cu, 1.80 μM Mo) (Tradecorp AZ; Guadalajara, Jalisco, Mexico). After a seven-day adaptation period, the nutrient solution was totally replaced and treatments were applied in the renewed nutrient solution.

Treatments to be tested consisted of 5, 10, and 15 μM V in the form of ammonium vanadate (NH_4_VO_3_) (Sigma-Aldrich; St. Louis, MO, USA). A control treatment was included which consisted of a nutrient solution without V. The pH of the solution was adjusted to 5.5 using concentrated H_2_SO_4_ and 1 N NaOH. The nutrient solution was aerated every 2 h for 15 min with an air pump (Hagen, Elite 802; Manfield, MA, USA), and along with the treatments, it was replaced every 7 d. Each container was provided with an air pump. A completely randomized experimental design was used where the experimental unit was represented by a single plant, with 12 replicates per treatment (details in S1 Fig). The experiment was done under greenhouse conditions at a mean temperature of 26°C, 60% relative humidity, and a photoperiod of 12 h light (300 μmol m^-2^ s^-1^ photon flux density) and 12 h darkness.

### Growth variables

Plant height and root length were recorded at 7, 14, 21, and 28 d after applying the treatments (dat). In the last evaluation (28 dat), the number of leaves and floral buds, stem diameter, root volume, leaf area, and fresh and dry biomass of roots, stems, leaves, and flowers were determined.

Plant height was measured from the base of the stem to the growth apex, while root length was measured from the base of the stem to the tip of the main root. Stem diameter was determined at the base using a digital vernier caliper (Truper 14388; Shanghai, China). Root volume was determined through the water displacement method, using a 15 mL graduated test tube. Leaf area was determined with a leaf area integrator (LI-CORLI-3000A; Lincoln, NE, USA). To determine the fresh and dry biomass weight, the plants were divided into roots, stems, leaves, and flowers and then weighed separately in an analytic scale (Adventurer Ohaus Pro AV213C; Parsippany, NJ, USA). The samples were subsequently placed in a forced air oven (Riossa HCF-125D; Guadalajara, Jalisco, Mexico) at 70°C. The weight of the dry biomass was determined 48 h later with the analytic scale.

### Concentration of chlorophylls *a*, *b*, and total in leaves and stems

Concentrations of chlorophylls (Chl *a*, Chl *b*, and Chl total) were determined through the method described by Geiger *et al*. [25]. To do this, 60 mg of macerated plant material was mixed with 1500 μL ethanol at 80% (v/v). The samples were incubated in a water bath (Thermo Fisher Precision; Waltham, MA, USA) at 80°C for 20 min, centrifuged at 14000 rpm in an Eppendorf 5424 centrifuge (Eppendorf, Germany) for 5 min, and the liquid phase was collected. This procedure was done twice more using ethanol at 80 and 50%, respectively. From the mixture collected from the triple ethanol extraction, 488 μL were taken and mixed with 1275 μL ethanol at 98% (v/v); this mixture absorbance was subsequently read in a spectrophotometer (Jenway 6715 UV/Vis; Staffordshire, UK) at 645 and 665 nm. Four independent biological replicates were done per treatment with two technical replicates. Chlorophylls *a* and *b* were determined with the following formulas:

Chlorophylla(μg/mgfreshweight)=(5.46xAbs665)‑(2.16xAbs645)

Chlorophyllb(μg/mgfreshweight)=(9.67xAbs645)‑(3.04xAbs665)

Total chlorophyll content was the sum of chlorophyll *a* and *b*. Additionally, we cross-checked through total chlorophyll analysis.

### Total amino acids in leaves, stems, and roots

The concentration of amino acids was quantified according to the ninhydrin method [26]. From the triple ethanol extraction, 500 μL were taken and mixed with 500 μL sodium citrate–ascorbic acid buffering solution (0.2% w/v), where the sodium citrate contained 16 mM citric acid and 34 mM sodium citrate at a pH of 5.2. Furthermore, 1000 μL ninhydrin (1% w/v) in ethanol at 70% (v/v) was added. The samples were incubated in a water bath at 95°C for 20 min and left to cool at room temperature. At the same time, the standard curve was prepared using leucine (10 mM in ethanol 70%). The samples were read in a spectrophotometer (Jenway 6715 UV/Vis) at 570 nm. Four replicates were done per treatment with three technical replicates.

### Total soluble sugars in leaves, stems, and roots

The total sugar concentration was determined in 0.5 g fresh plant material. The extraction was done in 50 mL ethanol 80% at constant boiling with occasional stirring, using a stirring hot plate with digital display (Corning PC-400D; New York, NY, USA) at 125°C. Samples were boiled for 25–30 min. Subsequently, the extracts were filtered and filled to a final volume of 20 mL. From this, 500 μL were taken and mixed with 500 μL ethanol 80%. The samples were placed on ice and 5 mL anthrone (Meyer; Queretaro, Mexico) was added, then they were placed in a water bath (Thermo Fisher Precision) at 95°C for 15 min; to finish the reaction, samples were placed on ice. The samples were read at 620 nm in a spectrophotometer (Jenway 6715 UV/Vis). To make the standard curve, glucose (Sigma-Aldrich; St. Louis, MO, USA) was used. Four replicates were done per treatment with two technical replicates.

### Concentrations of mineral nutrients and vanadium in leaves, stems, and roots

The concentrations of N, P, K, Ca, Mg, Fe, Cu, Zn, Mn, B, and V were determined in the dry biomass of leaves, stems, and roots. The N concentration was done through the semimicro-Kjeldahl method [27], while the concentrations of P, K, Ca, Mg, Fe, Cu, Zn, Mn, B, and V were determined through wet digestion of the dry material with a mixture of perchloric and nitric acid [28]. The obtained extracts were read in an inductively coupled plasma optical emission spectrometry equipment (Agilent 725 ICP-OES; Mulgrave, Victoria, Australia).

### Statistical analysis

The data were subject to a Shapiro-Wilk and Bartlett test (*P* ≤ 0.05) to prove normality and homogeneity of the variances. A logarithmic transformation was done when necessary; the data are shown without transformation. Once these assumptions were proven, a one-way analysis of variance (ANOVA) was done. Additionally, when there were differences, a mean separation was done through the Duncan method at a significance level of 0.05 (α = 0.05). All the analyses were done with the SAS software [29].

## Results

### Pepper plant growth and development are stimulated by vanadium

**Fig 1:**
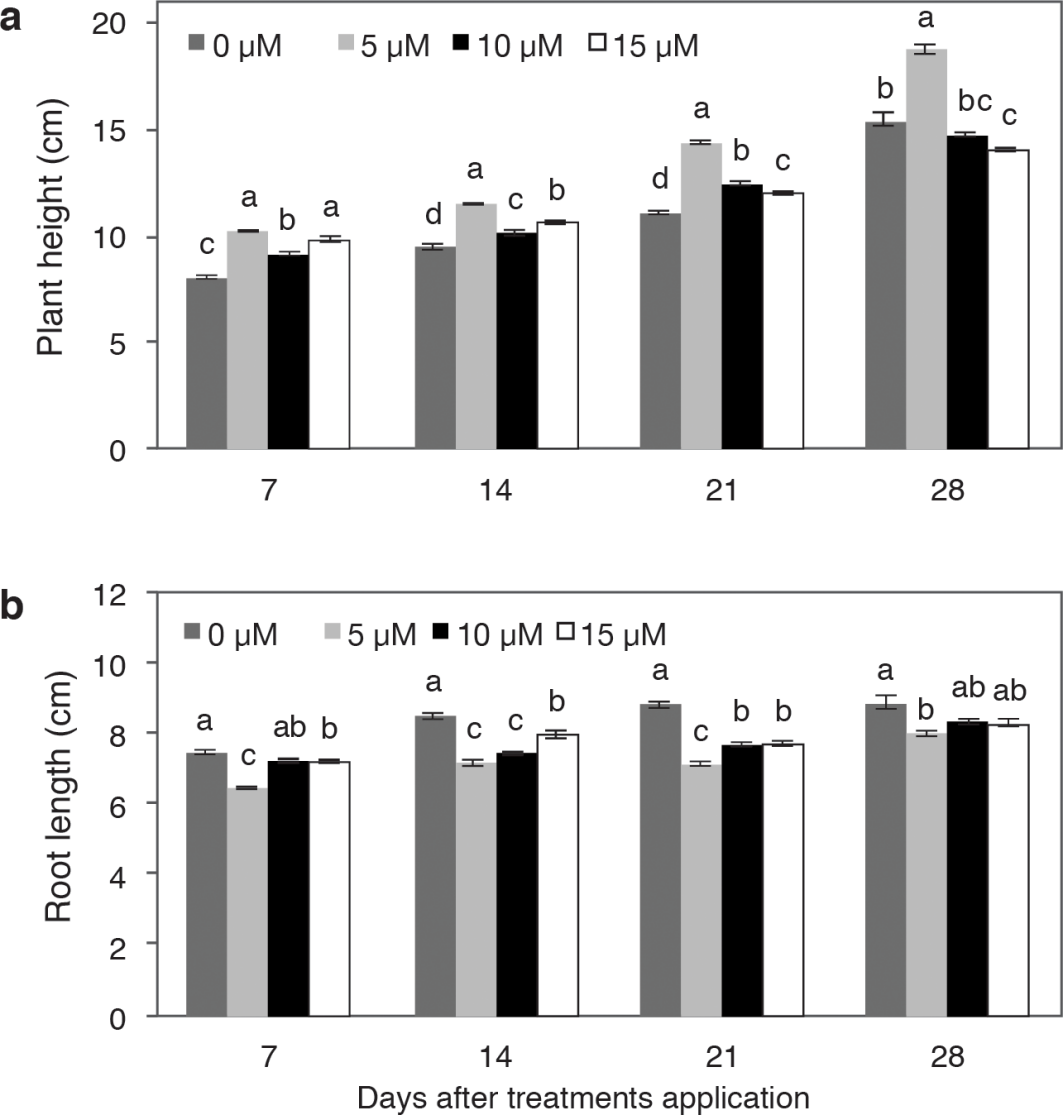
Effect of vanadium (0, 5, 10 and 15 μM V) on pepper plant growth. Plant height (a) and root length (b). Values are means ± standard error (SE) from at least five individual plants. Different letters above the bars indicate significant differences (Duncan, α = 0.05).

The experiment started with 37-d-old pepper cv. Mysterio F1 plants. They were treated with 0, 5, 10, and 15 μM V (Fig 1A). Seven days after the application of the treatments (dat), plant height was greater in the plants treated with 5 and 10 μM V than with the other treatments. After 14 dat, the plants treated with 5 μM V were taller than those of all the other treatments; at 28 dat, these plants were 21.7% taller than those in the control. Control plants were also lower than those treated with 10 and 15 μM V up to the third evaluation after applying the treatments (21 dat). At 28 dat, the plants treated with 15 μM V were lower than those in the control.

**Fig 2:**
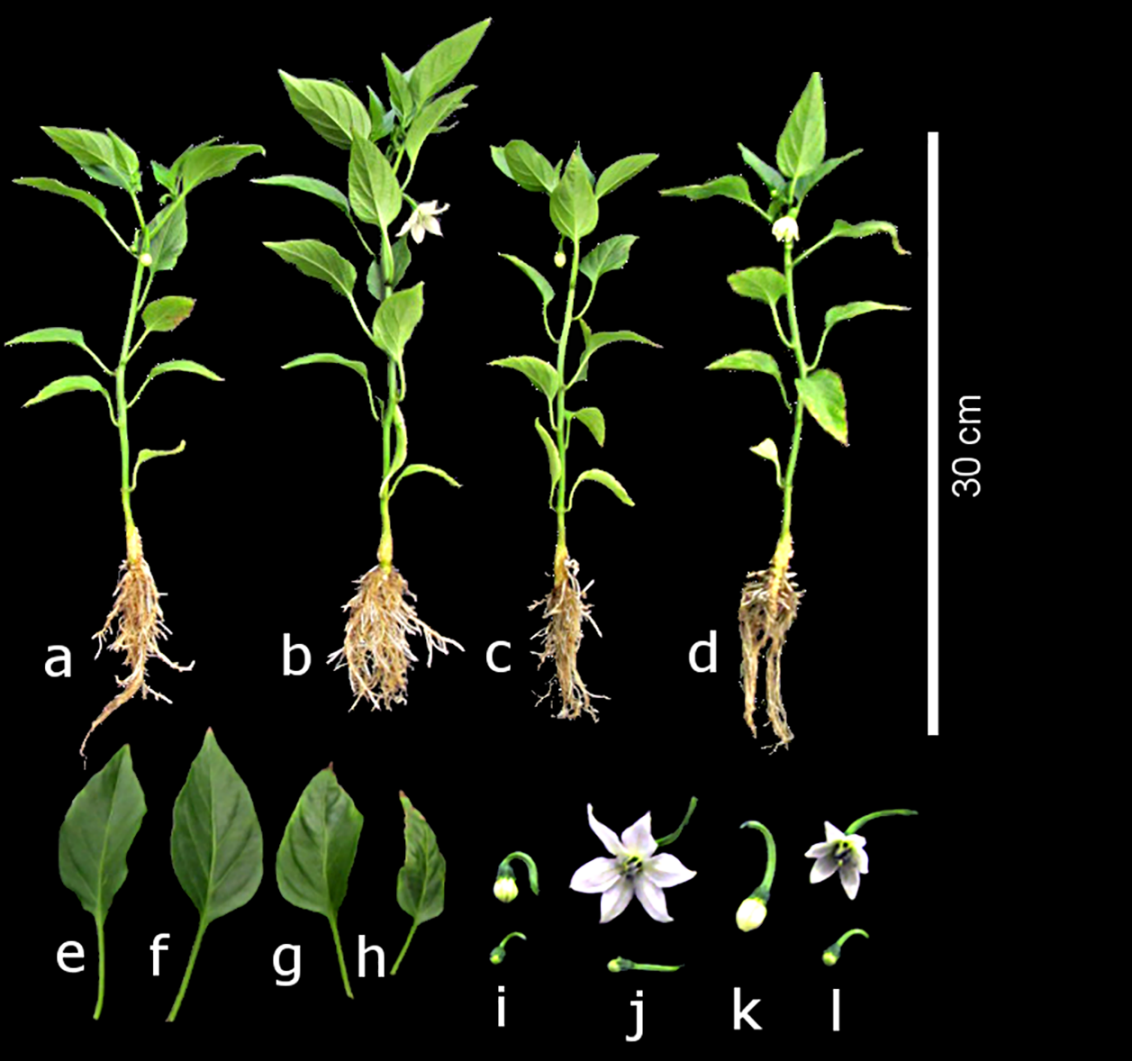
Growth and development displayed by pepper plants 28 days after treatment with different concentrations of vanadium (0, 5, 10 and 15 μM V). Control: a, e, i; 5 μM V: b, f, j; 10 μM V: c, g, k; and 15 μM V: d, h, l.

Vanadium had a different effect on root length and stem height. In all the measurements taken, control plants showed a higher value of these variables, while the plants treated with 5 μM V generally showed lower means (Fig 2A, 2B, 2C and 2D). Plants treated with 10 and 15 μM V showed intermediate values (Fig 1B). In general, as the V concentration increased, root growth increased, although V did not stimulate greater growth than the control.

**Fig 3:**
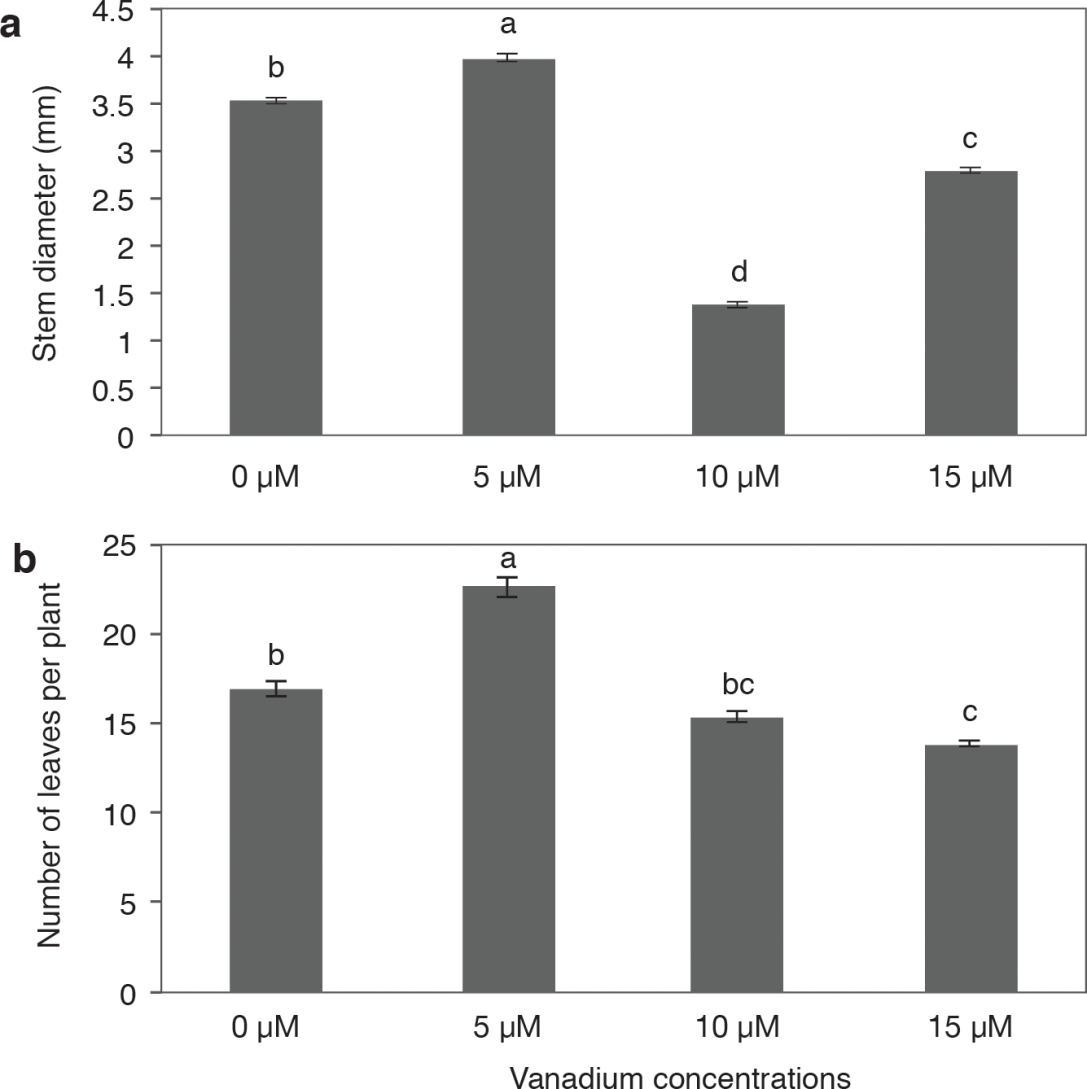
**Stem diameter (a) and number of leaves (b) of pepper plants grown in nutrient solutions containing different concentrations of vanadium (0, 5, 10 and 15 μM V) during 28 d**. Values are means ± standard error (SE) from at least five individual plants. Different letters above the bars indicate significant differences (Duncan, α = 0.05).

Stem diameter was greater in plants treated with 5 μM V, while those treated with 10 and 15 μM recorded the lowest values (Fig 3A). Likewise, the number of leaves per plant was higher with the application of 5 μM V, surpassing the control by 33.9%. Plants treated with 10 μM V showed no statistical differences with the control; however, the application of 15 μM V did decrease the number of leaves per plant by 18.6% with respect to the control. In general, there was a tendency of the number of leaves to decrease as the V concentration in the growth medium increased (Fig 3B).

The treatments with 5 and 10 μM V increased root volume by 40.9 and 54.5%, respectively, with regard to the control plants (Table 1). The plants treated with 15 μM V showed a root volume similar to that of the control. Despite the roots of the plants treated with 5 μM V being shorter than those of the control, there was a greater number of secondary roots, thus obtaining greater volume. The same tendency was observed with 10 μM V. However, when the V dose reached 15 μM, it was not different from the control (Table 1; Fig 2A, 2B, 2C and 2D).

**Table 1:** Root volume, leaf area, floral buds, and weight of fresh and dry flower biomass in pepper plants grown during 28 days in nutrient solutions containing different concentrations of vanadium (V).

Vanadium treatments	Root volume (mL)	Leaf area (cm^2^)	Number of floral buds	Weight of fresh flower biomass (mg)	Weight of dry flower biomass (mg)
Control (0 μM V)	2.2 ± 0.06b	50.85 ± 1.19a	3.5 ± 0.11b	131.25 ± 8.41b	17.72 ± 1.67b
5 μM	3.1 ± 0.11a	52.92 ± 3.51a	7.6 ± 0.19a	351.25 ± 6.40a	55.10 ± 4.11a
10 μM	3.4 ± 0.15a	54.73 ± 3.87a	4.1 ± 0.18b	148.75 ± 12.95b	22.67 ± 1.90b
15 μM	2.4 ± 0.13b	29.19 ± 0.99b	3.6 ± 0.2 b	158.50 ± 9.85b	33.35 ± 2.60b
*P*	0.0034	0.0206	<0.0001	<0.0001	<0.0020

Values are means ± standard error (SE) from at least five individual plants.

Different letters in each column indicate significant differences among treatments for each variable analyzed (Duncan, α = 0.05).

Leaves of plants treated with 15 μM V were smaller than those of control plants (Fig 2E, 2F, 2G and 2H). This finding was reflected in leaf area, since plants treated with 15 μM V obtained the lowest value of this variable. Plants exposed to 5 and 10 μM V showed no significant differences in comparison to the control. The number of floral buds per plant and the weight of fresh and dry flower biomass were higher with the application of 5 μM V than with the other treatments. The treatments with 10 and 15 μM showed statistically similar means as the control. It is important to note that at 24 dat, plants treated with V flowered faster than control plants. At 28 dat, the flowers of the 5 μM V treatment were larger and the number of floral buds higher. With the application of 10 μM V the floral buds were larger than those of control plants, although there was no flower development. The application of 15 μM V also promoted flower budding, but it was less abundant than that observed in the plants treated with 5 μM V. In the control plants, some floral buds appeared, but no flower opening was observed (Fig 2I, 2J, 2K and 2L). Nonetheless, floral buds looked normal and no aberrant phenotypes were observed among treatments. We did not measure flower fertility, since we aimed to evaluate the effect of V on the first growth stages of pepper plants.

**Fig 4:**
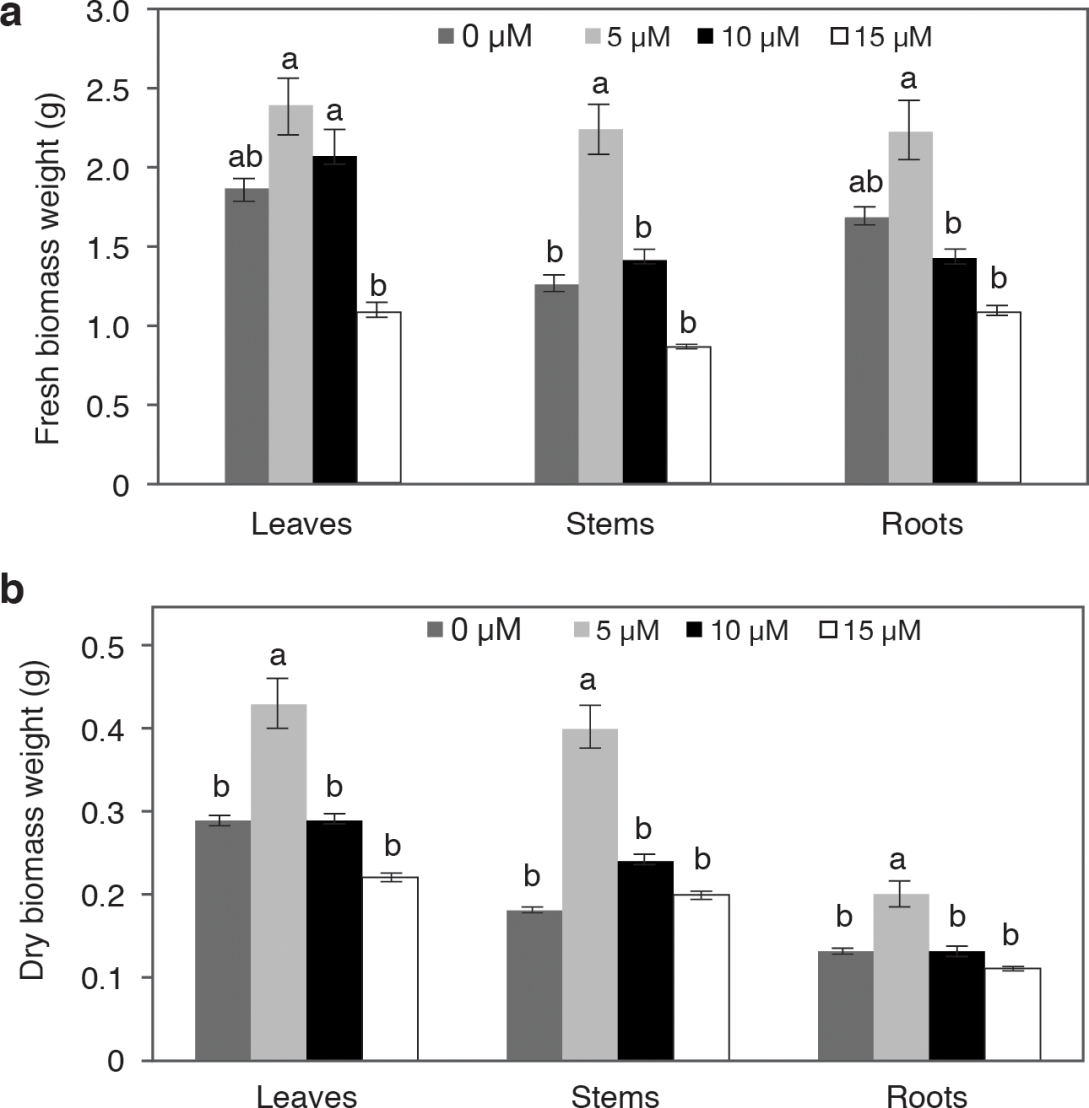
**Fresh (a) and dry (b) biomass weight of leaves, stems and roots of pepper plants grown in nutrient solutions containing different concentrations of vanadium (0, 5, 10 and 15 μM V) measured 28 days after treatment application**. Values are means ± standard error (SE) from at least five individual plants. Different letters above the bars for individual plant parts indicate significant differences (Duncan, α = 0.05).

The weight of fresh leaf biomass in plants treated with 5 and 10 μM V statistically similar to that of the control plants; with 15 μM V, a lower weight was observed. Similarly, the application of 5 μM V caused the highest weight in fresh stem biomass with respect to the rest of the treatments, including the control. On the other hand, there were no differences between the control, 10, and 15 μM V in the weight of fresh stem biomass. A similar behavior was observed in the weight of fresh root biomass, except that in this case there were no statistical differences between 5 μM V and the control. In general, there was a similar tendency in the three plant organs, since the weight of the fresh biomass decreased as the applied V concentration increased (Fig 4A). A similar effect was observed in the weight of dry biomass, where the application of 5 μM caused the highest means in the weight of leaves, stems, and roots, while there were no statistical differences between the control and plants treated with 10 and 15 μM V (Fig 4B). It is important to note that the weight of fresh and dry stem biomass showed the greatest differences between the control plants and the plants treated with 5 μM V; the treatment with 5 μM V more than doubled the control.

### Concentrations of chlorophylls in leaves and stems

The concentration of chlorophyll *a* in leaves was higher in plants treated with 5 μM V, followed by the control and in lower proportion by plants treated with 10 and 15 μM V. Contrarily, in stems, the highest concentration of chlorophyll *a* was found with the application of 15 μM V, followed by 5 μM V and in lower concentration by the 10 μM V and control treatments. The concentration of chlorophyll *b* in leaves and stems was the highest in the plants treated with 15 μM V. Particularly, the concentration of chlorophyll *b* was similar in the leaves of the control plants and those treated with 15 μM V. In stems, the lowest concentration of this pigment was observed with 5 μM V. The concentration of total chlorophylls in leaves was highest in the plants treated with 5 μM V, while the control and 15 μM were not statistically different. The lowest concentration of these molecules was found in plants treated with 10 μM V. In stems, the concentration of total chlorophylls was higher in the treatment with 15 μM, while the rest of the treatments were statistically similar. Additionally, the chlorophyll *a*/*b* ratio in leaves was the highest with 5 μM V, and decreased as the V concentration increased. Similarly, the chlorophyll *a*/*b* ratio in stems was the highest in plants treated with 5 μM, and the lowest ratio was obtained in the control and in plants treated with 10 μM V (Table 2).

**Table 2:** Concentration of chlorophyll (mg g^-1^ fresh biomass) in leaves and stems of pepper plants exposed to different concentrations of vanadium (V) in the nutrient solution for 28 days.

Chlorophyll	Plant part	Vanadium concentration				*P*
		Control (0 μM V)	5 μM V	10 μM V	15 μM V	
Chlorophyll*a*	Leaves	1513.73 ± 8.5b	1670.79 ± 6.1a	1455.36 ± 11.9c	1495.70 ± 6.0bc	<0.0001
	Stems	348.95 ± 3.7c	416.85 ± 5.5b	358.50 ± 3.5c	481.88 ± 3.5a	<0.0001
Chlorophyll *b*	Leaves	291.82 ± 6.2a	236.12 ± 4.1b	248.11 ± 4.7b	297.63 ± 3.2a	0.001
	Stems	112.65 ± 1.7b	49.44 ± 0.5c	107.33 ± 1.8b	127.55 ± 1.2a	<0.0001
Total Chlorophylls	Leaves	1805.55 ± 12.9b	1906.91 ± 8.3a	1703.47 ± 11.2c	1793.32 ± 9.0b	0.0002
	Stems	461.60 ± 4.3b	466.30 ± 5.9b	465.84 ± 5.0b	609.43 ± 4.6a	<0.0001
Chlorophyll *a/b* ratio	Leaves	5.21 ± 0.1c	7.10 ± 0.04a	5.89 ± 0.10b	5.03 ± 0.03c	<0.0001
	Stems	3.10 ± 0.05c	8.43 ± 0.06a	3.34 ± 0.04c	3.77 ± 0.01b	<0.0001

Values are means ± standard error (SE) from at least five individual plants. Different letters in each row indicate significant differences among treatments for each molecule and plant part analyzed (Duncan, α = 0.05).

### Concentration of total free amino acids and total soluble sugars

**Fig 5:**
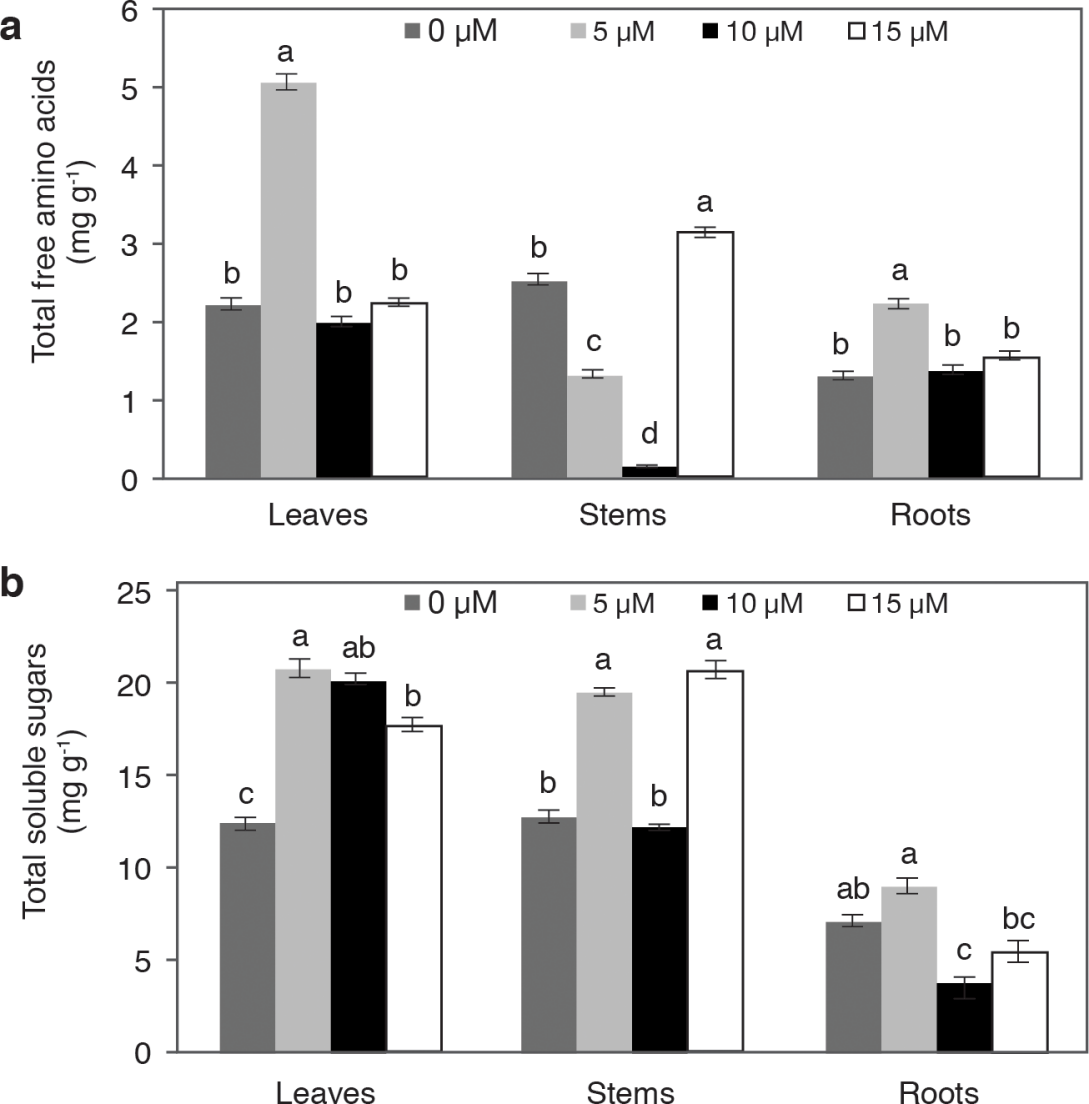
**Concentrations of total free amino acids (a) and total soluble sugars (b) in leaves, stems and roots of pepper plants grown in nutrient solutions containing different concentrations of vanadium (0, 5, 10 and 15 μM V) during 28 d**. Values are means ± standard error (SE) from at least five individual plants. Different letters above the bars for the individual plant parts indicate significant differences (Duncan, α = 0.05).

The concentration of amino acids in leaves and roots was higher in plants treated with 5 μM V, surpassing the control by 28.9% and 74.2%, respectively. The application of 10 and 15 μM V yielded statistically similar means to those of the control. A contrary effect was observed in stems, since the highest amino acids concentration was observed when applying 15 μM V, surpassing the control by 24.1%; with 5 and 10 μM V, the concentration of amino acids was lower than the control (Fig 5A).

In leaves, the highest concentration of sugars was obtained in plants treated with 5 and 10 μM V, followed by those treated with 15 μM; the lowest concentration of these molecules was observed in control plants. In stems, the application of 5 and 15 μM increased sugar content, surpassing the control by 51.9% and 61.6%, respectively. The application of 5 μM V had no effects on the concentration of sugars in the roots. Conversely, the treatments with 10 and 15 μM V had lower means than the control (Fig 5B).

### Mineral nutrient concentration in leaves, stems and roots

In leaves, V did not affect the concentrations of N, P, Ca, Fe, Cu, Zn, and B (Table 3). The K concentration was lower in all three treatments with V in comparison to the control. On the other hand, the Mg and Mn concentrations were only statistically lower in plants treated with 15 μM V in comparison to the control.

**Table 3:** Concentrations of mineral nutrient in leaves, stems and roots of pepper plants exposed to different concentrations of vanadium (V) for 28 days.

Vanadium (μM)	N	P	K	Ca	Mg	Fe	Cu	Zn	Mn	B
Leaves	g kg^-1^ DBW					mg kg^-1^ DBW				
0	34.9 ± 1.4a	4.4 ± 0.1a	12.8 ± 0.2a	12.6 ± 0.8a	6.6 ± 0.1a	158.6 ± 3.8a	36.0 ± 1.5a	48.1 ± 0.8a	662.2 ± 10.0a	67.6 ± 1.1a
5	50.2 ± 9.5a	3.7 ± 0.1a	9.8 ± 0.2b	12.4 ± 0.6a	6.4 ± 0.1a	142.2 ± 6.3a	45.6 ± 3.5a	41.7 ± 0.1a	711.2 ± 16.1a	59.9 ± 1.8a
10	25.2 ± 1.5a	3.6 ± 0.2a	8.5 ± 0.4b	11.1 ± 0.6a	5.9 ± 0.2ab	145.1 ± 9.6a	48.4 ± 1.5a	40.0 ± 1.9a	688.2 ± 35.4a	58.0 ± 2.2a
15	25.6 ± 0.7a	3.4 ± 0.3a	6.3 ± 0.5c	9.4 ± 0.8a	4.9 ± 0.4b	164.9 ± 17.6a	43.0 ± 5.9a	39.0 ± 3.8a	443.7 ± 34.9b	57.0 ± 4.3a
Stems	g kg^-1^ DBW					mg kg^-1^ DBW				
0	12.7 ± 0.6b	1.0 ± 0.1c	3.5 ± 0.2b	2.1 ± 0.1b	1.4 ± 0.1c	196.7 ± 17.0a	3.1 ± 0.3b	12.6 ± 0.9a	79.2 ± 2.8b	16.3 ± 1.1b
5	26.3 ± 2.90a	2.4 ± 0.1a	8.0 ± 0.2a	3.8 ± 0.1a	2.4 ± 0.1a	58.5 ± 1.9a	14.8 ± 1.2a	16.7 ± 1.9a	185.4 ± 4.4a	28.5 ± 0.9a
10	12.7 ± 0.9b	1.9 ± 0.1b	6.5 ± 0.4a	3.6 ± 0.2a	2.1 ± 0.1ab	99.3 ± 11.9a	13.9 ± 2.8a	14.6± 0.5a	171.3 ± 12.0a	32.5 ± 0.3a
15	12.6 ± 0.9b	1.3 ± 0.1c	4.0 ± 0.5b	2.5 ± 0.2b	1.6 ± 0.2bc	62.9 ± 5.5a	6.4 ± 0.9ab	13.1 ± 0.7a	103.5 ± 9.1b	21.2 ± 1.3b
Roots	g kg^-1^ DBW					mg kg^-1^ DBW				
0	29.4 ± 1.8a	1.4 ± 0.2b	3.0 ± 0.4ab	0.9 ± 0.1b	0.6 ± 0.1b	159.8 ± 13.9ab	7.9 ± 0.9b	20.2 ± 3.0ab	174.6 ± 25.9ab	9.7 ± 1.1b
5	26.7 ± 1.0a	0.7 ± 0.1b	1.3 ± 0.6b	0.8 ± 0.1b	0.4± 0.1b	76.2 ± 4.9b	4.8 ± 0.5b	13.4 ± 0.6b	77.7 ± 9.4b	5.3 ± 0.4b
10	38.6 ± 5.6a	1.4 ± 0.1b	2.3 ± 0.2ab	1.0 ± 0.1b	0.6 ± 0.1b	126.7 ± 13.7ab	7.3 ± 0.5b	19.9 ± 1.4ab	73.0 ± 5.1b	8.8 ± 0.8b
15	30.6 ± 1.0a	3.0 ± 0.5a	5.5 ± 0.9a	2.1 ± 0.3a	1.4 ± 0.2a	220.5± 21.5a	18.4 ± 3.1a	42.0 ± 6.0a	234.1 ± 39.1a	21.4 ± 2.7a

Values are means ± standard error (SE) from at least five individual plants. Means with different letters in each column indicate significant differences among treatments for each plant part analyzed (Duncan, α = 0.05). DBW: Dry Biomass Weight.

In stems, the N concentration was higher with the application of 5 μM V, while 10 and 15 μM yielded statistically similar means as the control (Table 3). The P and Mg concentrations were higher in plants treated with 5 μM V, and decreased as the V dose increased, until being similar to the control. The K, Ca, Cu, Mn, and B concentrations were higher with the application of 5 and 10 μM V, while there were no significant differences between plants treated with 15 μM and the control. V did not alter the concentrations of Fe and Zn in this plant organ.

In roots, the application of V did not affect the N concentration, but the P, Ca, Mg, Cu, and B concentrations were the highest with 15 μM V (Table 3). Interestingly, we observed a tendency for the concentrations of these nutrients to increase as the V dose in the nutrient solution increased. The concentrations of K, Fe, Zn, and Mn were similar between the control and the three V treatments, especially in plants treated with 5 and 15 μM V. Moreover, there was a clear tendency of these last nutrients to increase as the applied V dose increased.

**Fig 6:**
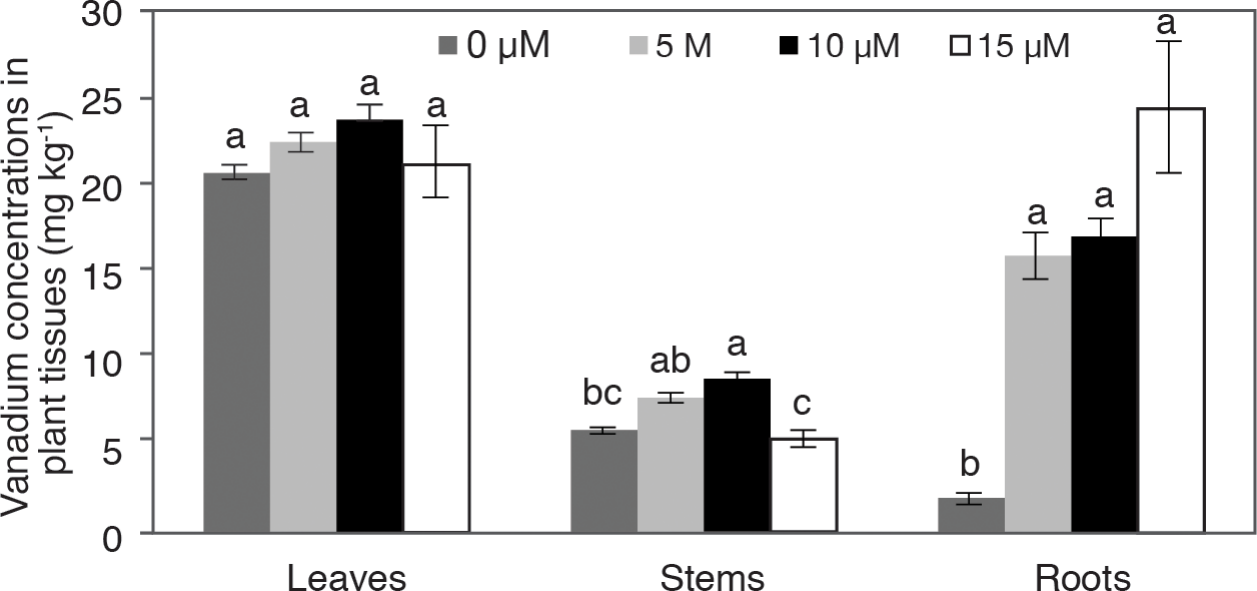
Vanadium (V) concentrations in leaves, stems and roots of pepper plants grown in nutrient solutions containing different concentrations of vanadium (0, 5, 10 and 15 μM V) during 28 days. Values are means ± standard error (SE) from at least five individual plants. Different letters above the bars for the individual plant parts indicate significant differences (Duncan, α = 0.05).

The concentration of V in leaves oscillated between 20.62 and 23.58 mg kg^-1^ dry biomass, with no observable significant statistical differences among treatments (Fig 6). In stems, the V concentration was higher in the treatments with 5 and 10 μM, while in the case of 15 μM the V concentration was similar to the control. The concentration of V in roots was significantly higher in plants treated with V than in the control; as the V dose increased, the V concentration increased from 8 to 13 times compared to the control.

## Discussion

### Vanadium stimulates pepper plant growth and development

Beneficial elements can positively influence plant growth, development, and production, even though they are not considered essential [30]. Each beneficial element can have specific functions and its effect can vary depending on different factors, including chemical form, dose, frequency of application, and genotypes in which they are applied. In general, beneficial elements can cause hormesis, a dose response phenomenon characterized by the stimulation of favorable mechanisms at low concentrations and inhibition or toxicity at high concentrations [31]. In the present work, we observed that during the first stages of plant growth, the application of 5 μM V stimulated plant height with respect to the control. However, at the end of the study (28 dat), plant height was similar between treatments with 10 and 15 μM V and the control (Fig 1A). In triticale (x *Triticosecale* Wittm.), the application of a high V dose (i.e. 120 μM) inhibited shoot growth [32], which coincides with our studies. The application of 5 μM V stimulated plant growth, with taller plants in all samplings. This could indicate that V is conferring greater elasticity to the tissue, providing greater water volume, which would be associated with cell expansion, giving plants greater growth [33]. Vanadium acts as a growth factor and influences reproduction, and it is metabolized utilizing the iron transport and the storage proteins transferrin and ferritin [34]. Vanadium's primary mode of action is as a cofactor that enhances or inhibits the enzymatic activity of vital proteins such as kinases and phosphatases [35], thus regulating plant growth and development.

Unlike plant height, root length was lower in plants treated with V in the measurements done at 7, 14, and 21 d, in comparison to the control. However, at 28 d, the root length of plants treated with 10 and 15 μM V was similar to the control (Fig 1B).

Considering only plants exposed to V (i.e. excluding the control), we observed a tendency to increase root length means as the V concentration increased and time passed. Similar results are reported in cuphea (*Cuphea viscosissima* x *C*. *lanceolata* ‘PSR 23’) [36], onion (*Allium cepa*) [37], and common bean (*Phaseolus vulgaris*) [38,39] when V was applied. Contrarily, root growth in rice was drastically decreased when 1 mM V was applied, while with 10 and 20 mM V, there was cell death in this organ [40]. In chickpea (*Cicer arietinum*), the application of 60 to 120 mg L^-1^ V resulted in root growth inhibition because of the stress caused by the doses [41]. The application of V in swamp morning glory (*Ipomoea aquatic*) caused the roots to decrease their growth and turn thicker and darker [42], which could be due to an accumulation of toxic pentavalent vanadium (V^+5^). This is the most toxic form of V, being more reactive with a number of essential enzymes [43], and affecting reproductive processes [44]. Vanadium acts as a phosphate analog and, as such, interferes with various vital enzymatic systems involved in phosphorus metabolism. Indeed, V may inhibit the activity of different ATPases, protein kinases, ribonucleases and phosphatases. Conversely, it may induce the activity of tyrosine kinase phosphorylase, NADPH oxidase, and adenylatecyclase [45]. Since V inhibits or stimulates the activity of many DNA or RNA enzymes, it may induce several genotoxic and mutagenic effects [46]. Hence, the effects of vanadium on various enzymes may be responsible for the diverse effects observed in living organisms exposed to this element. However, little information is available regarding the mechanism of V toxicity *in vivo*.

The application of 5 μM V gave off thicker stems as well as a greater number of leaves per plant with respect to the control. Conversely, the application of 10 and 15 μM V decreased the values of these variables (Fig 3A and 3B). In rice, the application of 1, 10, 20, and 40 mg L^-1^ V did not affect plant height or stem diameter, although with the application of 80 mg L^-1^ V, growth was restricted [21]. In Chinese green mustard (*B*. *campestris* ssp. *chinensis* var. *parachinensis*), the application of 1–80 mg L^-1^ V decreased plant height and number of leaves as the V level increased [22]. In lettuce (*Lactuca sativa*), root and leaf growth was inhibited by doses of 0.2 to 1.0 mg kg^-1^ V, while yields decreased with increasing rates of V. Toxicity symptoms in roots consisted of color darkening, club shape of the main roots, reduction of secondary root number and length, and necrosis. Leaves from plants treated with 0.5 and 1.0 mg kg^-1^ V also showed loss of turgidity [47]. Furthermore, lettuce plants treated with V displayed decreased growth, necrosis, and mild chlorosis, while at the lowest nutrient level (1 kg ha^-1^ 20-20-20 N-P-K), leaf discoloration, abnormal leaf growth (e.g., twisting and wrinkling), and lightened veins were observed in some plants [48]. The decrease in leaf growth might be related with the decrease in activity of the enzymes nitrate reductase and transaminase, involved in the synthesis of amino acids [49]. Moreover, V interferes in the activity of other enzymes, vital in the metabolism of living organisms [45], which could cause delayed growth. In the present study, the plants treated with 10 and 15 μM V (the highest doses tested) had smaller stem diameter and lower number of leaves in comparison to the other treatments.

Although root length was shorter in the treatments with V (Fig 1B), the root volume was greater at 5 and 10 μM V treatments (Table 1), which could be because there was a higher number of secondary roots than in the control plants (Fig 2A, 2B, 2C and 2D). This phenomenon may be attributed to a possible hormetic effect of the element on mechanisms controlling plant growth. In *Arabidopsis* plants treated with 25 μM V, root length was similar to the control, but there was greater formation and density of root hairs [50]. On the other hand, the application of 15 to 153 μM V inhibited the formation of secondary roots in cuphea [36]. Furthermore, the application of 160 to 400 μM V in bean plants grown hydroponically caused lesser growth of the main root and a lower number of secondary roots [39]. In the present study, the plants treated with the highest V concentration (15 μM) had a lower number of secondary roots, resulting in a lower root volume, in comparison to the rest of the treatments. Leaf area showed no significant effects from the treatments with 5 and 10 μM V), but with 15 μM V, the leaves were clearly smaller (Fig 2E, 2F, 2G and 2H). In common bean, the leaf length and leaf area decreased significantly as the V concentration increased starting at 240 μM V [39]. In chickpea, the application of 170 to 1180 μM V caused leaf deformities and size decrease as the concentration of applied V increased [51].

Besides increasing plant height, stem diameter, and number of leaves, the application of 5 μM V stimulated both the formation of floral buds (Table 1) and flower development (Fig 2I, 2J, 2K and 2L). When treatments with 10 and 15 μM V were applied, there were no statistical differences with the control. Similar results are reported in tomato, since the application of 250 ng V mL^-1^ caused taller plants, more leaves, and more flowers [11]. Furthermore, the higher production of fresh and dry biomass was also obtained with 5 μM V in leaves, stems, and roots, with no differences observed in plants treated with 10 and 15 μM, and the control. Contrasting results were obtained in the weight of dry biomass of soybean plants (*Glycine* max) stems and roots, since these plants significantly decreased when V concentration exceeded 30 mg kg^-1^ in the soil solution [52]. In Chinese green mustard, the weight of fresh root biomass decreased with the increase in the concentration of the applied V [23]. In chickpea, the weight of fresh root biomass decreased significantly with the application of 25 mg L^-1^ V [53]. Other studies have also reported a decrease in fresh and dry plant biomass with the application of V [3,32,36]. On the contrary, in soybean plants, the application of V in soils amended with manure caused an increase in fresh and dry biomass, since the reaction with the soil organic matter was capable of reducing V^+5^ to V^+4^ [54]. In rice, the higher weight of fresh and dry stem biomass was found in plants treated with 10 mg L^-1^ V [21]. In basil, the fresh and dry biomass of leaves and stems was not affected by the application of up to 40 mg L^-1^ V, while the root biomass increased linearly with increasing concentrations of vanadium (5–40 μM V) [55]. These results partly back up the role of V as a beneficial element in the growth and development of cultivated species, used in an appropriate dose.

### Vanadium differentially stimulates chlorophyll concentration in leaves and stems

The concentrations of chlorophyll *a* and total chlorophylls in leaves were higher with 5 μM V and lower with 10 and 15 μM V, with respect to the control. In stems, the concentrations of chlorophyll *a*, *b*, and total chlorophylls were higher in plants treated with 15 μM V; the concentrations of chlorophylls in the other treatments were lower or equal to the control (Table 2). Some of these results are similar to those obtained in swamp morning glory with the application of 0.5 to 2.5 mg L^-1^ V in a liquid medium in hydroponics, since as the V concentrations increased, the concentrations of chlorophyll *a* and *b* decreased [42]. Likewise, in chickpea, the concentrations of chlorophyll *a* and *b* decreased as the levels increased (15 to 120 mg L^-1^ V) [41]. In common bean, a decrease in the number of chloroplasts was reported in cells treated with V at concentrations of 240 and 320 μM [39]. In the unicellular green algae *Scenedesmus obliquus* and *Chlorella pyrenoidosa*, the application of 20 μM L^-1^ V stimulated growth and the formation of protoporphyrin-IX, essential precursor of chlorophylls [56]. Likewise, in maize leaves, the contents of chlorophyll *a* and *b* increased notably when the V concentration increased from 0 to 6.25 mg L^-1^ [10]. Similarly, the application of 250 ng mL^-1^ V increased the concentration of chlorophyll in tomato, which improved the Hill reaction in the chloroplasts and accelerated photosynthesis and plant development [11]. The increase in the concentration of chlorophylls in the present study could have been one of the mechanisms that induced greater plant growth, possibly due to a higher photosynthetic rate.

### Low concentrations of vanadium enhance the concentration of amino acids and sugars in leaves, stems, and roots

The concentration of amino acids in leaves and roots was higher in plants treated with 5 μM V, while with 10 and 15 μM there were no significant differences with respect to the control. In stems, the free amino acids were more abundant in plants treated with 15 μM V, while with 5 and 10 μM V they were less so than the control (Fig 5A). In sugar beet (*Beta vulgaris* L. subsp. *vulgaris* var. *altissima*), the application of 10 mM V caused toxic effects, decreasing leaf growth and the concentrations of chlorophylls and amino acids [48]. In *Arabidopsis*, it has been proven that a single amino acid can stimulate (i.e. histidine) or inhibit (i.e. tyrosine) flowering [57], thus the fluctuations in the metabolism of these molecules could have determining effects on this reproductive process. In the present study, plants treated with 5 μM V flowered earlier and produced a higher concentration of total free amino acids in leaves and roots as compared to the other treatments. In leaves, the concentration of total soluble sugars was higher in all the treatments with V. In stems, control plants and those treated with 10 μM had the same concentration of sugars; these values were statistically lower than those found in plants treated with 5 and 15 μM (Fig 5B). In sugar beet, the sucrose content increased by 28% in plants treated with 10 mM V, compared with the control [48]. A higher sucrose content could come from a higher fixation ratio of carbon dioxide, which could have stimulated growth.

### Vanadium differently affects the mineral nutrient status of pepper plants

Vanadium did not affect the N concentration in leaves and roots, while in stems the N concentration was two times higher in the treatment with 5 μM V, as compared to the control and the rest of the treatments. In common bean, the application of 3 and 6 mg kg^-1^ V increased N concentration in roots and leaves [38]. Likewise, soybean plants treated with 0.5 and 1.0 mg kg^-1^ V showed a higher N content [54]. The P concentration was differentially affected by V. In leaves, there were no observable changes in the different treatments tested. In stems, P was statistically higher with 5 μM V, and decreased as the V concentration increased until it was similar to the control. The opposite behavior was observed in roots, where the P concentration increased as the level of V was increased, until reaching double that of the control. Partially similar results were reported in common bean leaves and roots, as there were no differences in the P concentration under the evaluated V treatments [39]. Conversely, in soybean plants, increasing levels of V (0–2.0 mg kg^-1^) decreased the P concentrations [52]. In swamp morning glory, the P concentration decreased in leaves, stems, and roots as the V concentration in the nutrient solution increased [39]. In chickpea, low concentrations of V promoted P absorption, while high levels of V inhibited the absorption of this nutrient [53]. In the present study, there was a positive relation between V and P, since by increasing the V dose in the nutrient solution, the P concentration in roots increased, and the lowest level of V (5 μM) yielded the highest P concentration in stems. The relation between P absorption and V is important due to their chemical analogy, since V inhibits the activity of enzymes in which P is an important component, like phosphatases, liases, synthases, and ATPases [58,59]. Moreover, the absorption of vanadates is determined by phosphate transports in the root [51]. Nevertheless, P replacement by V does not always inhibit the enzymatic activity, and the identification of the V binding to different enzymes has been of great importance in comprehending, recognizing, and evaluating new protein structures [15].

Increasing levels of V decreased the concentration of K in pepper leaves, while in stems the concentration of K was more than double with the 5 μM V treatment than in the control, which subsequently decreased as V increased until it was statistically equal to the control. In roots, there were no statistical differences with the control. In other crops like rice [10], soybean [38,54], and basil [55], no significant effects of V on K concentrations in leaves, stems or roots have been documented. However, in the present study, plants treated with 15 μM V were shorter and leaves displayed some necrosis (Fig 2), which is a typical symptom of K deficiency. This coincides with the decrease in the K concentration found herein.

Calcium concentration in leaves was not affected by the application of V. In stems, 5 and 10 μM V favored Ca concentration. In roots, the application of 15 μM V increased Ca while in the rest of the treatments it remained similar to the control. In soybean roots, the application of 3 and 6 mg kg^-1^ increased the Ca concentration; however, in leaves, the application of V decreased this variable [38]. In basil, the Ca concentration was not affected by V in leaves and stems; in roots, the application of 5 to 40 mg L^-1^ V decreased the Ca levels [55].

Magnesium concentration decreased significantly with 15 μM V in leaves, while in stems the concentration of this macronutrient increased by 70 and 50% with the application of 5 and 10 μM V, respectively. In roots, the application of 15 μM V increased Mg concentration by over 130%. Opposite results were found in soybean, as V decreased the Mg concentration in roots and did not affect the leaves [38]. In basil, V did not affect the Mg concentration in leaves and stems, but like in soybean, the Mg concentration in roots decreased [55], which is contrary to what was found in the present study.

The concentrations of micronutrients like Fe and Zn were not affected by V in leaves or stems, compared to the control. Likewise, no differences among treatments were observed in roots, though the application of 10 μM V favored the concentrations of Fe and Zn. Similarly, V did not exert any effect on micronutrient concentrations in soybean [38]. In pennyroyal (*Mentha pulegium*), the concentrations of Fe and Zn decreased in leaves and stems with the application of V, while in roots the Fe concentration increased and Zn concentration was not altered [12]. In basil leaves, the Fe concentration decreased as the level of V increased, but the Zn concentration was not changed. No effects were reported in stems with regard to Fe or Zn, while in roots increasing V doses gradually decreased the FE and Zn concentrations [55].

Vanadium concentrations tested in the present study did not affect the concentrations of Cu and B in leaves. In stems, the concentration of these two elements increased with the application of 5 and 10 μM V. In roots, the concentrations of Cu and B only increased with 15 μM V. Similar results were observed in the B concentration in higher leaves of soybean, while the Cu concentration was not affected by V in roots and leaves [54].

In the present study, the Mn concentration in leaves decreased by 33% with 15 μM V. In stems, this variable increased by more than double with the 5 and 10 μM V treatments. In roots, there were no significant differences between treatments. In soybean roots and leaves, there were also no significant changes in Mn concentrations from the application of V [38]. In pennyroyal, the application of 5 mg L^-1^ decreased the Mn concentrations in both leaves and stems, but there were no effects from V in roots [12]. In basil leaves, stems, and roots, no changes were reported in the Mn concentrations as a result of V applications [55].

The V concentration in leaves was statistically similar in all treatments, including the control. In stems, the V concentration increased as the applied dose increased, while in roots the V concentration was drastically superior, compared to the control. In pennyroyal [12] and basil [56], V concentrations in leaves were similar to those of the control with the application of 5, 10, and 20 mg L^-1^ V. In stems, V concentration decreased as the applied V dose increased, contrary to what happens in the roots. In general, there was a higher V concentration in leaves, followed by roots, and less so in stems (leaves>roots>stems). These results differ from those reported in pennyroyal (roots>stems>leaves) [12], soybean (roots>leaves) [38], common bean (roots>leaves) [39], swamp morning glory (roots>leaves>stems) [42], and basil (roots>leaves>stems) [55], where a higher concentration of V was reported in roots in comparison to leaves. These findings indicate that different plant species have different V absorption and mobilization capacities, as well as diverse response mechanisms to the same stimulus (V). However, further studies are still required in order to elucidate the physiological, biochemical, and molecular mechanisms that are activated in response to the application of different V concentrations and sources, in different plant genotypes. As a biostimulant that can cause hormetic effects in plant and animal cells, it is important to study the levels of accumulation that this element can reach in edible organs and determine the thresholds between its beneficial and toxic properties. Moreover, given its therapeutic, metabolic, and enzymatic importance, future studies should focus their efforts on assessing its potential in biofortification processes of important agricultural crops. In any case, the impact of V on the environment and the levels of accumulation that it can reach in soil and water must be determined to ensure its proper use.

While V did not significantly affect nutrient status in leaves (Table 3), the biomass production was enhanced, especially when applying 5 μM V (Fig 4). Coincidently, concentrations of total free amino acids and total soluble sugars also increased in this plant part in V-treated plants (Fig 5). Furthermore, chlorophylls *a* and total also increased with the application of 5 μM V (Table 2). The beneficial role of V appears to be related to chlorophyll concentration, possibly through an effect on iron nutrition [60,61]. Under our experimental conditions, plants exposed to 5 μM V exhibited higher concentrations of chlorophyll *a* and total, though no changes in Fe concentrations were found among treatments for this plant part (Table 3). Interestingly, V has been shown to be a metabolic inhibitor of ATPases in cells, which could interfere with active transporters and hence the uptake of other essential elements. Indeed, the uptake of Mn and Cu is stimulated by the presence of V [62], which was observed in our study in stems, but not in leaves (Table 3).

Just recently, it was demonstrated that the application of plant growth biostimulants based on amino acids improved yield and grain quality of winter wheat [63]. Amino acids are the basic building blocks of proteins and fulfill multiple functions in the plant, including structural, metabolic and transport roles [64]. In fact, amino acids can act as stress-reducing agents and a source of nitrogen and hormone precursors [65,66,67]. Nevertheless, whether V affects hormone biosynthesis and growth through a direct stimulation of amino acids production under our experimental conditions deserves further studies.

Soluble sugars act on the supply of carbohydrates from source organs to sink ones. Sucrose and hexoses may both upregulate growth-related genes and downregulate stress-related genes [68]. Therefore, further molecular, biochemical and physiological studies are needed to uncover signaling components triggered by V under our experimental conditions, and to determine specificity and cross-talk during plant growth and development stimulated by V through soluble sugar biosynthesis and accumulation in different plant tissues.

## Conclusions

The application of 5 μM V increased plant height, stem diameter, number of leaves and floral buds, root volume, and weight of fresh and dry biomass of pepper plants. However, at higher applied concentrations (i.e. 10 and 15 μM V), this element had negative effects on the plant. Moreover, the application of this element at low concentrations stimulated the concentration of chlorophylls in the leaves, as well as amino acids and sugars in leaves and roots. Negative effects of V were only found in the K concentrations in leaves, while high levels of V (15 μM) negatively modified the concentrations of Mg and Mn in leaves. Other nutrients, like Fe and Mn, were not changed in any of the organs evaluated. The rest of the essential nutrients analyzed in the different plant organs had a synergic effect with V, especially with 5 μM in stems and 15 μM in roots. Also, the low concentration of V (5 μM) accelerated the flowering process and increased the number of floral buds. Given the stimulating effect of V on pepper plant growth and development during the vegetative stage and beginning of flowering, the present study proves that V can function as a beneficial element and have potential use improving the production of agricultural crops. Physiological, biochemical, and genomic approaches would further elucidate novel mechanisms of action and support the extensive use of V in plants, as well as its potential use in biostimulation and biofortification strategies. These possibilities await further studies.

## Supporting information

S1 Fig Containers with a capacity of 35 L containing the nutrient solutions with different concentrations of vanadium (0, 5, 10 and 15 μM V). (DOCX)
