# Supplementary material for: Vanadium stimulates pepper plant growth and flowering, increases concentrations of amino acids, sugars and chlorophylls, and modifies nutrient concentrations
Source: PLoS One. 2018 Aug 9;13(8):e0201908. doi: 10.1371/journal.pone.0201908 (PMC6085002; doi:10.1371/journal.pone.0201908)
Supplement: S1 Fig — (DOCX) [file pone.0201908.s001.docx]

**Vanadium stimulates pepper plant growth and flowering, increases concentrations of amino acids, sugars and chlorophylls, and modifies nutrient concentrations**

Atonaltzin García-Jiménez, Libia Iris Trejo-Téllez, Dagoberto Guillén-Sánchez, Fernando Carlos Gómez-Merino*


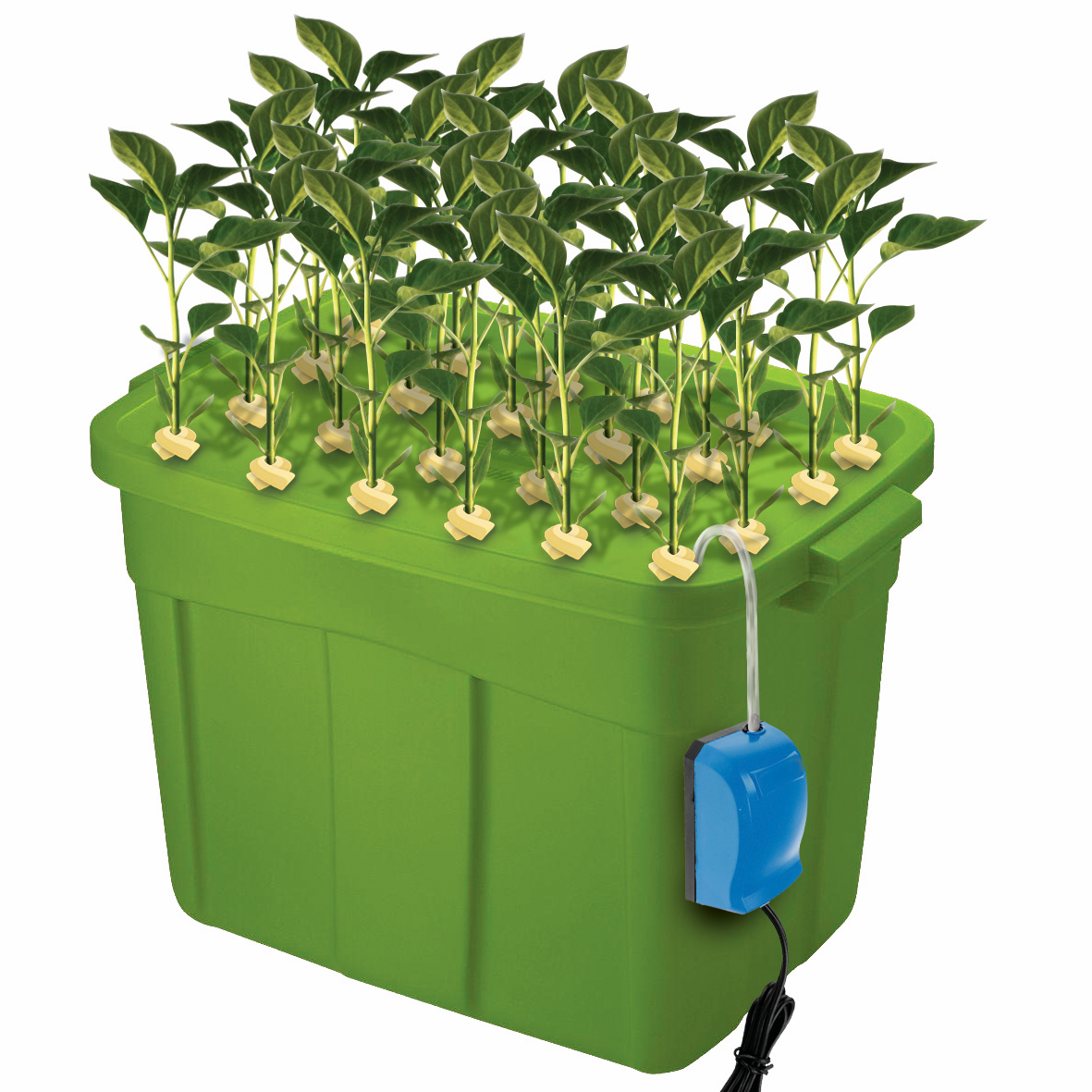


**S1 Figure** Containers with a capacity of 35 L containing the nutrient solutions with different concentrations of vanadium (0, 5, 10 and 15 µM V). Pepper plants were placed into the holes of the perforated covers of the containers , and gently winded with sponge around the stems. Each container was provided with an air pump in order to aerate the nutrient solution, as described in Materials and Methods.
